# Co-production of ethanol and squalene using a *Saccharomyces cerevisiae ERG1* (squalene epoxidase) mutant and agro-industrial feedstock

**DOI:** 10.1186/s13068-014-0133-7

**Published:** 2014-09-24

**Authors:** Claire M Hull, E Joel Loveridge, Nicola J Rolley, Iain S Donnison, Steven L Kelly, Diane E Kelly

**Affiliations:** Institute of Life Science, College of Medicine, Swansea University, Swansea, Wales SA2 8PP UK; School of Chemistry, Cardiff University, Cardiff, Wales CF10 3AT UK; Institute of Biological, Environmental & Rural Sciences, Aberystwyth University, Gogerddan, Aberystwyth, Wales SY23 3EE UK

**Keywords:** Bio-based products, *ERG1*, Ethanol, Sterol, Squalene, Squalene epoxidase

## Abstract

**Background:**

Genetically customised *Saccharomyces cerevisiae* that can produce ethanol and additional bio-based chemicals from sustainable agro-industrial feedstocks (for example, residual plant biomass) are of major interest to the biofuel industry. We investigated the microbial biorefinery concept of ethanol and squalene co-production using *S. cerevisiae* (strain YUG37-*ERG1*) wherein *ERG1* (squalene epoxidase) transcription is under the control of a doxycycline-repressible *tet0*_*7*_*-CYC1* promoter. The production of ethanol and squalene by YUG37-*ERG1* grown using agriculturally sourced grass juice supplemented with doxycycline was assessed.

**Results:**

Use of the *tet0*_*7*_*-CYC1* promoter permitted regulation of *ERG1* expression and squalene accumulation in YUG37-*ERG1,* allowing us to circumvent the lethal growth phenotype seen when *ERG1* is disrupted completely. In experiments using grass juice feedstock supplemented with 0 to 50 μg doxycycline mL^−1^, YUG37-*ERG1* fermented ethanol (22.5 [±0.5] mg mL^−1^) and accumulated the highest squalene content (7.89 ± 0.25 mg g^−1^ dry biomass) and yield (18.0 ± 4.18 mg squalene L^−1^) with supplements of 5.0 and 0.025 μg doxycycline mL^−1^, respectively. Grass juice was found to be rich in water-soluble carbohydrates (61.1 [±3.6] mg sugars mL^−1^) and provided excellent feedstock for growth and fermentation studies using YUG37-*ERG1*.

**Conclusion:**

Residual plant biomass components from crop production and rotation systems represent possible substrates for microbial fermentation of biofuels and bio-based compounds. This study is the first to utilise *S. cerevisiae* for the co-production of ethanol and squalene from grass juice. Our findings underscore the value of the biorefinery approach and demonstrate the potential to integrate microbial bioprocess engineering with existing agriculture.

## Background

Microbial biotechnology is employed for the generation of novel industrial, pharmaceutical and medical compounds and assists in the development of more efficient commercial production processes. Microorganisms that possess the enzymatic machinery needed to unlock fuel energy from cellulosic and lignocellulosic fractions of plant biomass [1-5] and recombinant strains that can utilise alternative substrates (such as inulin [6]) for the production of additional bio-based products [7-10], are of major interest to biofuel and biorefinery industries. In the following study we investigated the potential to co-produce ethanol and squalene using a genetically customised strain of *S. cerevisiae.*

Squalene is a polyunsaturated, triterpenic hydrocarbon (2,6,10,15,19,23-hexamethyltetracosa-2,6,10,14,18,22-hexaene) with nutritional, cosmetic, pharmaceutical and medical applications [11-14]. As a key intermediate of bacterial hopanoid and eukaryotic sterol biosynthesis [15,16], squalene is ubiquitous in nature. Squalene can be derived from plant oils [17,18] and the liver oil of deep sea sharks [19,20]. However, given the increasing commercial demand for squalene alongside growing international concern for the fate of food crops and the exploitation of marine habitats, sustainable sources of squalene are required.

**Figure 1:**
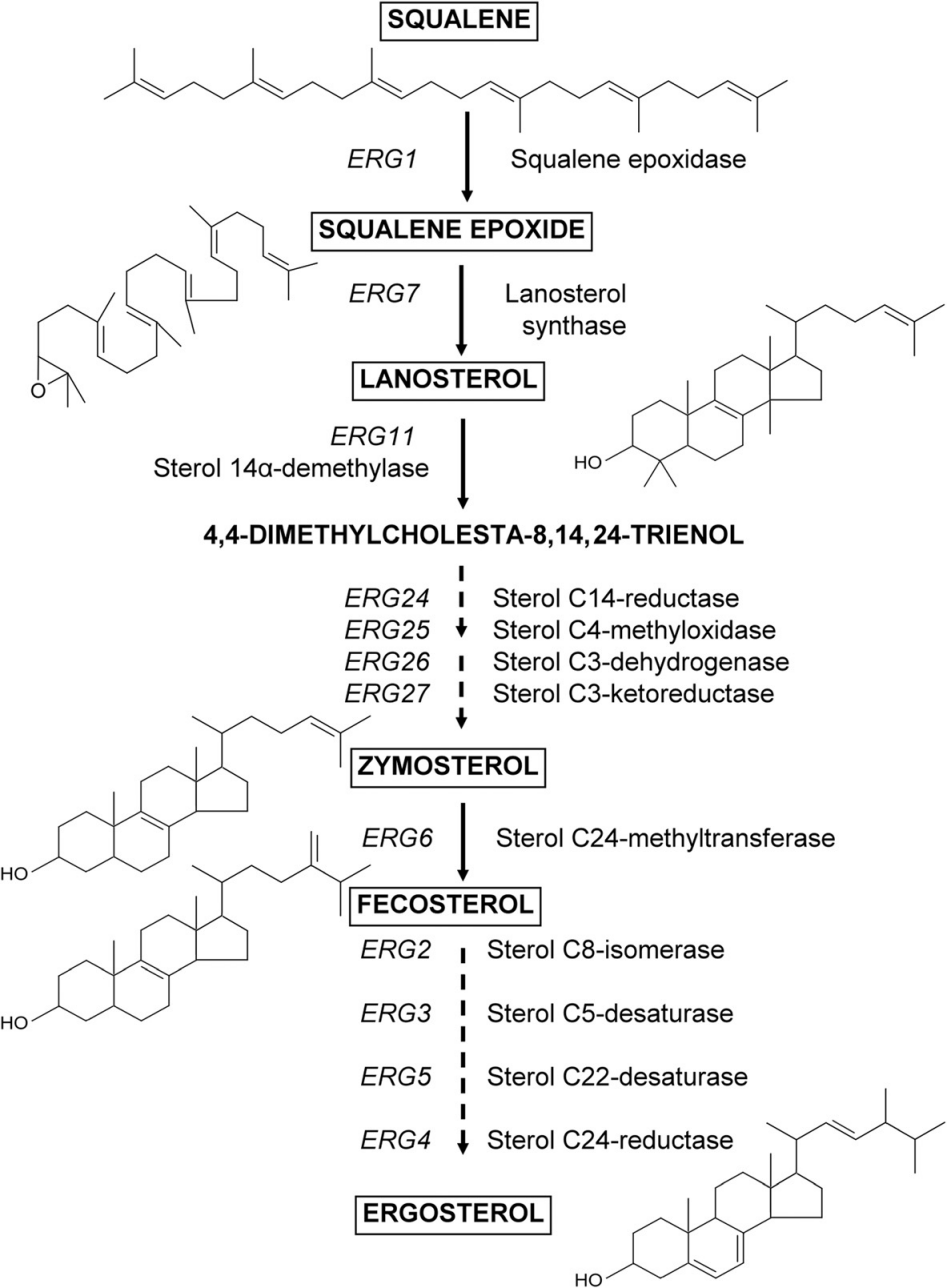
**Ergosterol biosynthetic pathway in yeast.** Structures of squalene and selected sterol intermediates (boxed); unbroken arrow = single enzymatic step; broken arrow = multiple enzymatic steps. Gene names are upper case, italicised; protein names are lower case, regular.

Research into the molecular controls and growth conditions that affect sterol biosynthesis [21-24] has highlighted the scope to utilise the brewing yeast *S. cerevisiae* for squalene production [25-27,16]. Under low oxygen or anaerobic conditions [28,29] and in heme-deficient yeast [30], squalene accumulates (≥70% of total squalene fraction) in intracellular lipid droplets [16,31]. However, under aerobic growth conditions squalene is converted to ergosterol through the action of proteins encoded by the *ERG* (ergosterol biosynthetic) genes [23]. Of these, squalene epoxidase, encoded by *ERG1* [32,33] is an oxygen-requiring enzyme [34] that is essential for the initial conversion of squalene to squalene epoxide (Figure 1).

We investigated the potential to produce squalene as a bio-based chemical product of yeast fermentation using a customised *S. cerevisiae* strain (YUG37-*ERG1*) wherein *ERG1* gene transcription is under the control of a doxycycline-repressible promoter that replaces the *ERG1* promoter at the chromosomal locus [35,36]. Because low growth temperature and decreased oxygen availability are favourable for both ethanol fermentation and the inhibition of yeast squalene epoxidase [29,34], we envisaged the opportunity to co-produce ethanol and squalene using a biorefinery approach. For this purpose we utilised juice extracted from perennial ryegrass (*Lolium perenne)* [37,38]. Grass juice represents one of several fractions from *L. perenne* biomass that are currently under investigation as feedstock for biofuel production and microbial bioprocess engineering in the United Kingdom [39-41]).

## Methods

### Yeast strains and growth media

Squalene production studies were undertaken using a laboratory strain of *Saccharomyces cerevisiae* (*YUG37-ERG1*) in which squalene epoxidase *(ERG1* protein*)* expression is controlled using a previously optimised doxycycline-repressible *tet0*_*7*_*-CYC1* promoter system [35,36,42]. The wild-type *S. cerevisiae* parent strain (YUG37; Hegemann, J., unpublished) was used as a comparator during initial experiments. Both strains were routinely maintained on yeast-peptone-dextrose (YPD) medium containing (w/v): 2% glucose, 2% Bacto Peptone and 1% yeast extract - including 2% agar when required (all media components supplied by Difco).

For ethanol and squalene co-production experiments, grass juice (GJ) feedstock was extracted from ryegrass *Lolium perenne* supplied by the Institute of Biological, Environmental Research and Rural Sciences (IBERS, UK) as described previously [37]. GJ was screened to remove large particulates, autoclaved (121°C, 30 min) and frozen (-80°C) prior to use as a growth and fermentation substrate. All other chemicals used in this study were supplied by Sigma unless otherwise stated.

### Gas chromatography-mass spectrometry (GC-MS) sterol analysis

Cell pellets from experimental cultures were resuspended in 7:3 methanol:water containing 18% (w/v) potassium hydroxide, 0.1% (w/v) pyrogallol and 10 μg cholesterol (as the internal standard), and heated at 90°C for 2 h. Non-saponifiable lipids (squalene and sterols) were extracted into glass HPLC vials using 3 × 2 mL hexane. Extracts were evaporated to dryness using a centrifugal evaporator (Heto Maxi dry plus) and derivatised using 100 μL N,O-bis(trimethylsilyl)trifluoroacetamide and trimethylchlorosilane (BSTFA-TMCS [99:1]) and 50 μL anhydrous pyridine at 70°C for 2 h [43]. Trimethylsilyl (TMS)-derivatised sterols were analysed using a 7890A GC-MS system (Agilent Technologies) with a DB-5MS fused silica column (30 m × 0.25 mm × 0.25 μm film thickness; J & W Scientific). The oven temperature was initially held at 70°C for 4 min, then increased at 25°C min^−1^ to a final temperature of 280°C, which was held for a further 25 min. Samples were analysed in splitless mode (1 μL injection volume) using helium carrier gas, electron impact ionization (ion source temperature of 150°C) and scanning from m/z 40 to 850 [44].

GC-MS data files were analysed using MSD Enhanced ChemStation software (Agilent Technologies Inc.) to determine squalene and sterol profiles for all isolates and for derivation of integrated peak areas. Sterols were identified by reference to retention times and mass fragmentation patterns for known standards.

### Sterol analysis of strains

Initial experiments were undertaken to determine the effect of doxycycline on the growth and sterol composition of the wild-type YUG37 parent and doxycycline-repressible YUG37-*ERG1* strain. Single colonies from each were used to inoculate 10-mL volumes of YPD medium (containing 0 to 50 μg doxycycline mL^−1^) with starting cell densities of 5 × 10^5^ mL^−1^. Cultures were grown in 50-mL flasks at 30°C, 180 rpm for 48 h, after which time the cell biomass was harvested by centrifugation. Cell pellets were dried to constant mass for biomass (g dry weight L^−1^) determinations, and the cellular squalene and sterol content determined by GC-MS as described above.

### Production of ethanol and squalene from GJ feedstock

#### Simultaneous co-production

Experiments to achieve simultaneous co-production of ethanol and squalene were performed in 100-well honeycomb microplates using a Bioscreen C (Oy Growth Curves Ab Ltd, Finland). Uniform starting (t_0_h) culture densities were achieved by resuspending a single YUG37-*ERG1* colony in GJ and diluting to obtain 5 × 10^5^ cells mL^−1^ in 1 mL of GJ containing 0 to 50 μg doxycycline mL^−1^. Starting cultures were vortexed and aliquoted into Bioscreen wells (3 × 300 μL replicates per doxycycline treatment). All experiments were incubated at 20°C (typical of ale production) in the Bioscreen (no shaking regime) for 96 h, with optical density readings (at 600 nm) taken every 45 min [31]. Data was exported from the Bioscreen in ASCII format prior to analysis using Excel (Microsoft Office 2003). Dry weight determinations and GC-MS sterol analyses were performed on the biomass fractions from pooled Bioscreen wells.

Growth parameters were derived as described previously [38]. Briefly, ΔOD values describe maximum OD minus minimum OD; the lag phase is defined as the length of time a culture spends at <10% of maximum OD; T_½_Max values are equivalent to the time taken to reach half the maximum increase in growth of a culture (ΔOD × 0.5). Minimum (that is, fastest) doubling times (DT_min_) were estimated by dividing the natural logarithm of 2 by the fastest culture growth rates (μ), where μ is the gradient of the linear trend line fitted to log-transformed OD data.

#### Sequential production

The stepwise production of ethanol and squalene was monitored using the Bioscreen. YUG37-*ERG1* was first grown for 48 h at 20°C using GJ feedstock; at t_48_h Bioscreen measurements were suspended and 100 μL of supernatant removed from experimental wells for ethanol analysis [38]. This volume was immediately replaced with 100 μL of fresh GJ containing doxycycline (to give a final concentration of 5 or 50 μg doxycycline mL^−1^) and the Bioscreen restarted using a medium shaking regime to promote new growth and squalene accumulation. Dry weight determinations and GC-MS sterol analyses were performed on the biomass fractions from pooled Bioscreen wells at t_96_h.

#### Sugar and ethanol assays

At specific time intervals (t_0_h, t_48_h, t_72_h and t_96_h) Bioscreen measurements were suspended and a 10-μL volume of culture supernatant removed from representative experimental wells for ethanol and sugar analyses.

Sugar analyses were performed on suitably diluted (typically 2,500-fold) culture medium in 100 mM potassium phosphate, pH 7.0, containing 10 mM MgSO_4_, 1 mM NAD^+^, 1.5 mM ATP and 20 U mL^−1^*Leuconostoc mesenteroides* glucose-6-phosphate dehydrogenase (Worthington Biochemical Corporation). Concentrations of glucose, fructose, sucrose and fructan were determined from the changes in absorbance at 340 nm following sequential addition of 20 U mL^−1^ *S. cerevisiae* hexokinase (Worthington Biochemical Corporation), 20 U mL^−1^*E. coli* phosphoglucose isomerase (Megazyme International Ireland Ltd), 1.5 U mL^−1^ *S. cerevisiae* sucrase/maltase (Megazyme International Ireland Ltd) and 10 U mL^−1^ fructanase from *Aspergillus niger* (Megazyme International Ireland Ltd), respectively. Standards of glucose, fructose, sucrose and chicory inulin were used to calibrate the assay.

Ethanol determinations were made using a spectrophotometric ethanol assay kit (K-ETOH 11/06; Megazyme Ltd) according to manufacturer’s instructions. All samples were diluted 1,000-fold with distilled water prior to analysis.

## Results and discussion

Of the many microorganisms that could be employed for ethanol production, *S. cerevisiae* remains the species of choice in industrial-scale fermentation processes [45]. Similarly, whilst squalene could be sourced from alternative microbes ([16,27] for reviews), because of the need for specific culture conditions and because many have not been granted GRAS (generally regarded as safe) status, they cannot be exploited in a commercial setting. The present study is the first to utilise *S. cerevisiae* for the co-production of ethanol and squalene from a single feedstock. We identify clear avenues for the integration of yeast biotechnology and existing agriculture for the production of bio-based compounds, thereby adding value to such fermentations.

### Regulation of growth and squalene accumulation

Data from initial experiments using YPD medium (Table 1) demonstrate the potential to maximise squalene production from *S. cerevisiae* through regulation of *ERG1* expression (Figure 1) and indicate that in order to achieve an optimal squalene yield, repression of *ERG1* gene expression must be sufficient to result in increased squalene accumulation, but not complete growth inhibition. The highest squalene content (7.85 ± 0.02 mg g^−1^ dry biomass) was recorded in YUG37-*ERG1* grown using YPD supplemented with 50 μg doxycycline mL^−1^; however, because the biomass of these cultures was low (1.39 ± 0.12 mg L^−1^), the squalene titre was sub-optimal (10.87 ± 0.93 mg L^−1^). Conversely, whilst the squalene content of YUG37-*ERG1* grown with 0.025 μg doxycycline mL^−1^ was comparatively lower (3.57 ± 0.2 mg g^−1^ dry biomass), higher overall culture growth (4.3 ± 0.28 mg L^−1^) supported an improved squalene titre (Table 1; 15.04 ± 1.42 mg L^−1^).

**Table 1:** **Phenotypic sterol analysis of YUG37 parent and YUG37-** ***ERG1*** **mutant**

**DOX**		**Sterol composition (mg sterol g** ^**−1**^ **)**				**Total**	**Biomass**	**Squalene titre**
**(μg mL** ^**−1**^ **)**	**Strain**	^**a**^ **14α-DM**	^**b**^ **14α-M**	**Ergosterol**	**Squalene**	**(mg g** ^**−1**^ **)**	**(g L** ^**−1**^ **)**	**(mg L** ^**−1**^ **)**
**0**	**YUG37**	1.45 [0.27]	0.33 [0.03]	3.27 [0.39]	0.16 [0.08]	5.31 [0.19]	3.75 [0.35]	0.50 [0.11]
	**YUG37-** ***ERG1***	1.51 [0.29]	0.39 [0.07]	3.08 [029]	0.40 [0.03]	5.28 [0.24]	4.55 [0.21]	1.76 [0.02]
**0.025**	**YUG37**	1.30 [0.16]	0.20 [0.06]	3.09 [0.35]	0.17 [0.13]	4.75 [0.02]	4.25 [0.21]	0.55 [0.31]
	**YUG37-** ***ERG1***	1.55 [0.09]	0.40 [0.03]	2.43 [0.08]	3.57 [0.20]	7.88 [0.31]	4.30 [0.28]	**15.04 [1.42]**
**0.05**	**YUG37**	1.47 [0.22]	0.26 [0.03]	3.03 [0.14]	0.30 [0.04]	4.98 [0.19]	3.90 [0.14]	1.23 [0.03]
	**YUG37-** ***ERG1***	0.78 [0.10]	0.40 [0.08]	2.20 [0.09]	4.24 [0.30]	7.55 [0.33]	2.50 [0.28]	10.31 [0.79]
**0.5**	**YUG37**	1.36 [0.22]	0.24 [0.06]	2.79 [0.32]	0.23 [0.06]	4.77 [0.30]	3.95 [0.07]	1.02 [0.14]
	**YUG37-** ***ERG1***	0.45 [0.01]	0.17 [0.02]	1.58 [0.12]	6.75 [0.20]	8.96 [0.06]	1.75 [0.21]	11.93 [1.27]
**5**	**YUG37**	1.54 [0.29]	0.29 [0.06]	3.06 [0.42]	0.20 [0.10]	5.03 [0.11]	3.60 [0.42]	0.57 [0.12]
	**YUG37-** ***ERG1***	0.21 [0.04]	0.08 [0.04]	1.49 [0.02]	7.66 [0.01]	9.45 [0.08]	1.48 [0.11]	11.30 [0.82]
**50**	**YUG37**	1.21 [0.23]	0.23 [0.01]	2.93 [0.26]	0.14 [0.06]	4.63 [0.27]	3.75 [0.35]	0.59 [0.05]
	**YUG37-** ***ERG1***	0.11 [0.03]	0.02 [0.01]	1.50 [0.03]	**7.85 [0.02]**	9.48 [0.10]	1.39 [0.12]	10.87 [0.93]

All cultures grown at 30°C, 180 rpm for 48 h on YPD medium. Mean values (n = 3 [±SD]); DOX = doxycycline. Maximum squalene content and yield are emboldened.

^a^ = sum of all 14α-demethylated sterols; ^b^ = sum of 14α-methylated sterols.

### Alterations in yeast sterol composition

**Figure 2:**
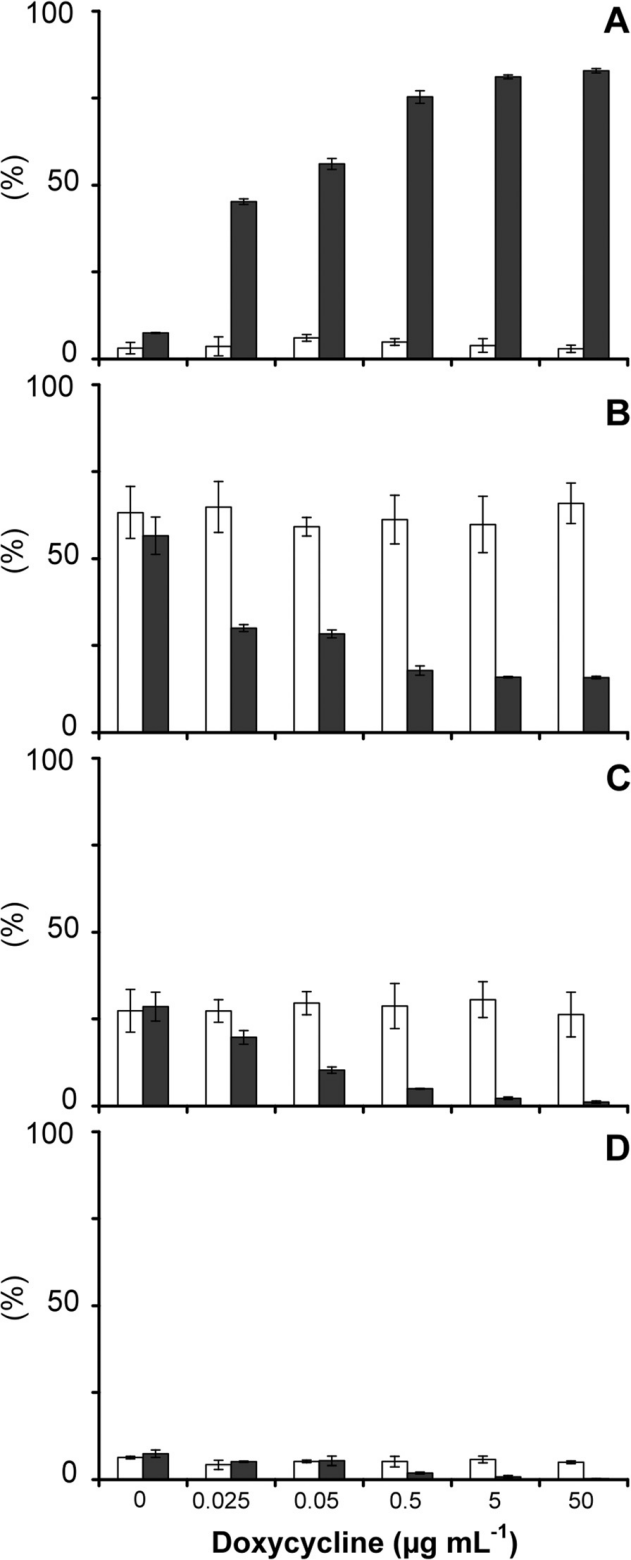
**Relative (%) abundance of sterols in YUG37 (open bars) and YUG37-** ***ERG1*** **(filled bars) cultured using YPD; mean values (n = 2 [±SD]). A** = squalene; **B** = ergosterol; **C** = sum of all 14α-demethylated sterols; **D** = sum of 14α-methylated sterols.

Doxycycline did not alter the sterol composition of the YUG37 parent across the range of doxycycline concentrations (0 to 50 μg mL^−1^) tested (Table 1). That no significant differences in the proportion of 14α-demethylated or 14α-methylated sterol intermediates were detected in treated YUG37 cultures (Figure 2C and 2D) indicates that doxycycline did not affect the function of other *ERG* genes or proteins involved in ergosterol biosynthesis (Figure 1). Changes in the sterol composition of doxycycline-treated YUG37-*ERG1* cultures, namely overall decreases in both 14α-demethylated and 14α-methylated sterol intermediates, are consistent with the specific inhibition of squalene epoxidase expression. Enhanced accumulation of squalene at the expense of other sterol intermediates has previously been reported in *S. cerevisiae* treated with the squalene epoxidase inhibitor, terbinafine [27].

### Growth and ethanol fermentation: grass juice feedstock

**Figure 3:**
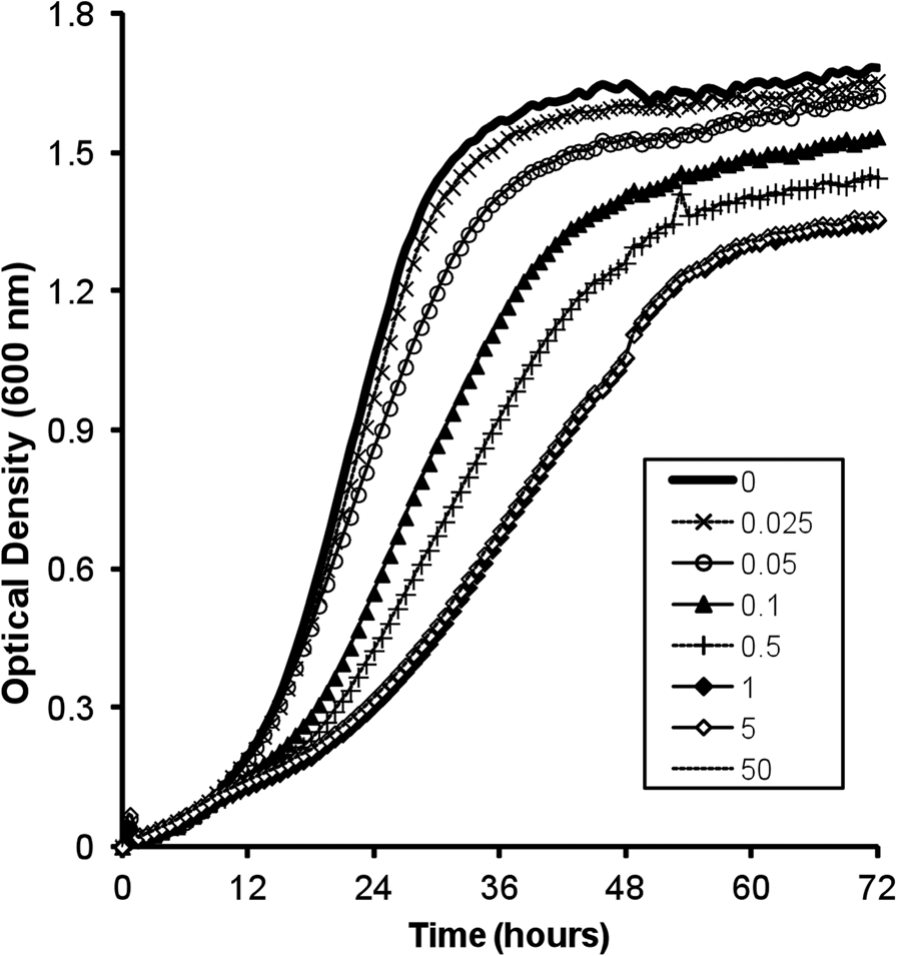
**Bioscreen growth curves for YUG37-** ***ERG1*** **at 20°C on GJ feedstock.** Legend indicates concentration of doxycycline (μg mL^−1^); curves for 5 and 50 μg mL^−1^ overlap.

Growth parameters for YUG37-*ERG1* grown using grass juice (GJ) (Table 2 and Figure 3) underscore data from previous studies that highlight its potential as a feedstock for *S. cerevisiae* [37,38]. GJ was rich in water-soluble carbohydrates (Table 3) that were readily utilised for growth and ethanol fermentation. In simultaneous ethanol and squalene production experiments*,* maximum concentrations of ethanol (20 to 23 mg mL^−1^) were produced after 72 h of fermentation. Similar concentrations (22.5 [±0.5] mg ethanol mL^−1^) were recorded in sequential production experiments (Table 4, asterisked data) after just 48 h, prior to the addition of doxycycline. Taken as a whole, the ethanol titres in this study are comparable to those achieved previously using GJ and alternative wild-type laboratory strains of *S. cerevisiae* [37,38].

**Table 2:** **Growth parameters for YUG37-** ***ERG1*** **cultured on GJ at 20°C in the Bioscreen**

**DOX**	**ΔOD**	**Lag phase (h)**	**T**_**½**_ **Max (h)**	**DT**_**min**_ **(h)**
**(μg mL** ^**−1**^ **)**				
**0**	1.69 [0.01]	11.3 [0.4]	21.8 [0.4]	3.8 [0.4]
**0.025**	1.66 [0.01]	11.4 [0.2]	22.6 [0.5]	4.3 [0.1]
**0.05**	1.63 [0.01]	11.5 [0.4]	24.1 [1.2]	5.4 [0.2]
**0.1**	1.54 [0.01]	11.5 [0.7]	28.9 [0.9]	6.0 [0.7]
**0.5**	1.43 [0.02]	11.8 [0.4]	31.5 [0.7]	8.6 [0.5]
**1**	1.34 [0.02]	11.5 [0.4]	35.8 [0.4]	10.3 [0.4]
**5**	1.35 [0.01]	11.5 [0.7]	38.0 [2.8]	10.3 [0.7]
**50**	1.34 [0.02]	10.9 [0.5]	37.0 [1.4]	10.4 [0.9]

Mean values (n = 3 [±SD]). DOX = doxycycline; ΔOD = maximum minus minimum optical density reading at 600 nm; Lag phase = length of time culture remains at < 10% of maximum OD; T_½_Max = time taken to achieve half maximal culture growth (maximum OD minus minimum OD × 0.5); DT_min_ = fastest observed doubling time.

**Table 3:** **Glucose, fructose, sucrose and fructan content of GJ media during fermentation with YUG37-** ***ERG1***

	**Content in medium (mg mL** ^**−1**^ **)**				
**Sugar**	**GJ t**_**0**_	**GJ t**_**48**_	**GJ t**_**96**_	**GJ** ^**0.5**^ **t**_**96**_	**GJ** ^**50**^ **t**_**96**_
**Glucose**	7.3 [1.8]	0.2 [0.2]	**—**	**—**	**—**
**Fructose**	22.2 [2.0]	0.4 [0.1]	0.5 [0.1]	0.8 [0.5]	0.6 [0.1]
**Sucrose**	0.5 [0.3]	0.2 [0.2]	0.1 [0.2]	0.2 [0.2]	0.7 [0.2]
**Fructan**	31.2 [2.4]	17.8 [0.5]	17.4 [0.04]	17.8 [1.2]	15.7 [2.9]
**Total**	61.1 [3.6]	18.5 [0.9]	18.1 [0.2]	18.9 [1.4]	16.9 [2.9]

Mean values (n = 2 [±SD]). Superscripts 0.5 and 50 refer to media containing 0.5 and 50 μg mL^−1^ doxycycline, respectively; Strikethrough = not detected.

**Table 4:** **Sterol composition, dry weight biomass and squalene titre of YUG37-** ***ERG1*** **cultured using GJ**

**DOX**	**Sterol composition (mg sterol g** ^**−1**^ **)**				**Total**	**Biomass**	**Squalene titre**
**(μg mL** ^**−1**^ **)**	^**a**^ **14α-DM**	^**b**^ **14α-M**	**Ergosterol**	**Squalene**	**(mg g** ^**−1**^ **)**	**(g L** ^**−1**^ **)**	**(mg L** ^**−1**^ **)**
**0**	1.09 [0.23]	0.47 [0.16]	3.51 [0.83]	0.19 [0.09]	5.25 [0.35]	4.80 [0.28]	0.88 [0.39]
**0.025**	0.97 [0.46]	0.27 [0.27]	2.64 [0.40]	3.98 [0.68]	7.85 [0.35]	4.50 [0.28]	**18.0 [4.18]**
**0.05**	0.73 [0.21]	0.28 [0.17]	2.00 [0.34]	5.14 [0.25]	8.15 [0.21]	2.25 [0.21]	11.5 [0.53]
**0.5**	0.31 [0.07]	0.09 [0.01]	2.63 [0.48]	5.92 [0.27]	8.95 [0.15]	1.78 [0.11]	10.5 [1.11]
**5**	0.10 [0.01]	0.01 [0.01]	1.27 [0.15]	**7.89 [0.25]**	9.28 [0.11]	1.44 [0.04]	11.4 [0.70]
**50**	**—**	**—**	1.31 [0.46]	**7.84 [0.25]**	9.15 [0.21]	1.45 [0.07]	11.4 [0.20]
**5	**—**	**—**	4.34 [0.07]	2.22 [0.28]	6.55 [0.35]	5.40 [0.14]	12.0 [1.83]
**50	**—**	**—**	3.80 [0.37]	2.45 [0.02]	6.25 [0.35]	5.20 [0.14]	12.7 [0.24]

All cultures maintained at 20°C in the Bioscreen. Mean values (n = 3 [±SD]); DOX = doxycycline. Maximum squalene content and titre are emboldened. Asterisks indicate sequential production experiments supplemented with additional GJ + DOX after 48 h growth in the absence of DOX.

Strikethrough = not detected.

^a^ = sum of all 14α-demethylated sterols; ^b^ = sum of 14α-methylated sterols.

### Ethanol titres

High ethanol titres have recently been achieved using pure inulin and soybean feedstock and an engineered yeast strain (*Saccharomyces* sp. W0) expressing the inulinase gene from *Pichia guilliermondii* [10]. Work is now needed to address the potential to use recombinant yeast to ferment grass juice to ethanol on an industrial scale and alternative (for example, flocculating [46-48] or high ethanol producing [10] host strains of *S. cerevisiae.*

### Squalene accumulation: grass juice feedstock

**Figure 4:**
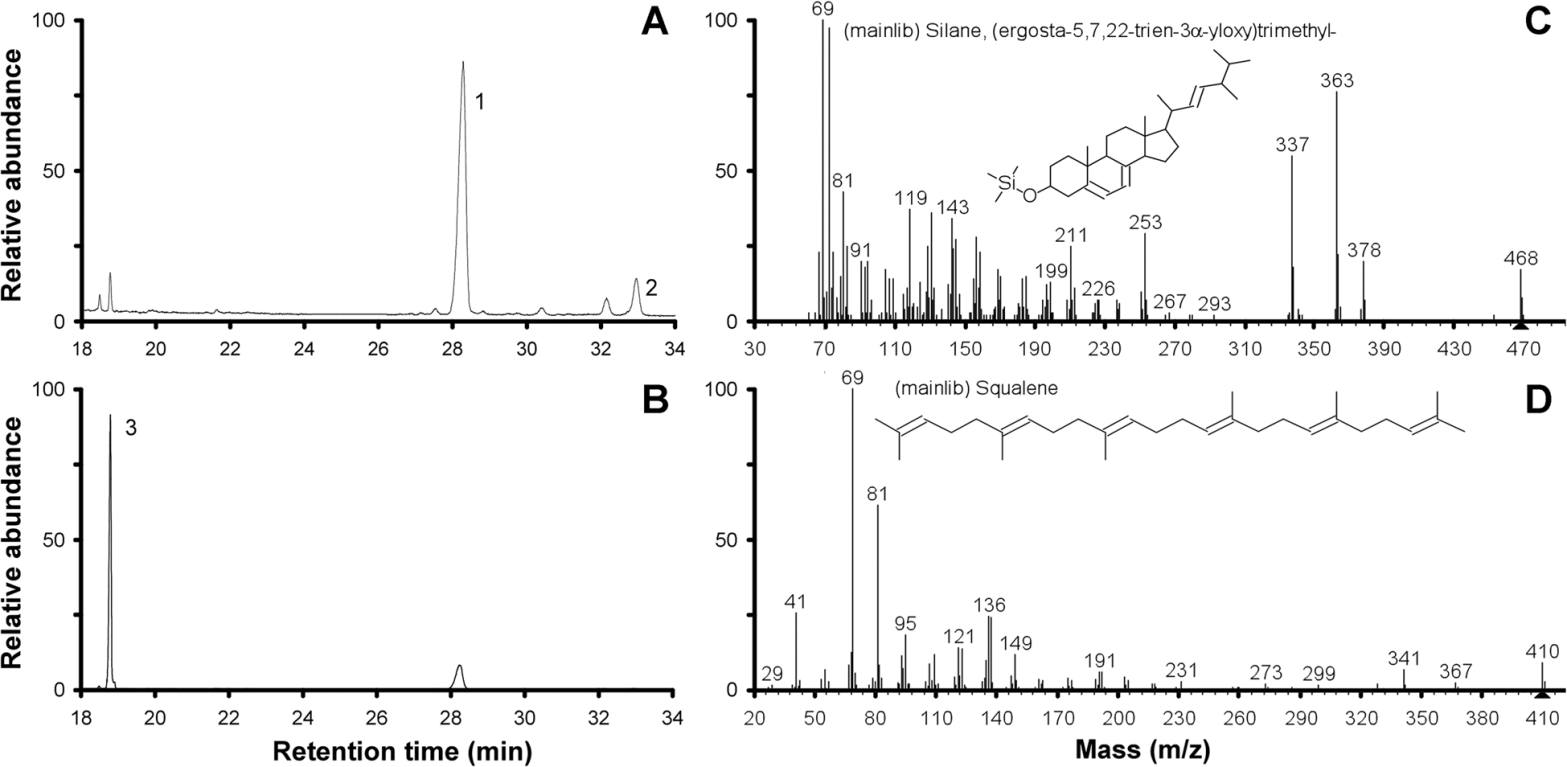
**GC-MS analysis of YUG37-** ***ERG1*** **. A)** and **B)** Total ion chromatograms for YUG37-*ERG1* grown on GJ and on GJ + 50 μg doxycycline mL^−1^, respectively. 1 = ergosterol; 2 = lanosterol; 3 = squalene. **C)** and **D)** Fragmentation patterns for TMS-derivatised ergosterol and squalene, respectively (MSD ChemStation NIST/EPA/NIH Mass Spectral Library Version 2.0).

The highest squalene content (7.89 ± 0.25 mg g^−1^ dry biomass) and squalene titres (18.0 ± 4.18 mg L^−1^) were achieved during simultaneous production experiments in which GJ was supplemented (at t_0_h) with 5 and 0.025 μg doxycycline mL^−1^, respectively (Table 4 and Figure 4). In subsequent experiments the highest total biomass (5.2 to 5.4 g L^−1^) was recorded 48 h after the removal of culture supernatant and the addition of fresh doxycycline-supplemented GJ (Table 4, asterisked data). However, concomitant with the accumulation of ergosterol during initial growth in the absence of doxycycline (4.34 ± 0.07 and 3.80 ± 0.37 mg ergosterol g^−1^ dry biomass) squalene titres (12.0 ± 1.83 and 12.7 ± 0.24 g squalene L^−1^, respectively) were lower than those recorded in simultaneous production experiments in which doxycycline was present from t_0_h. The use of bioreactors to maintain optimal growth and fermentation conditions for ethanol and squalene co-production using YUG37-*ERG1* is now an avenue for bioprocess development and commercial scale-up.

### Advantages of the doxycycline-regulatable promoter system

The maximum squalene titres achieved using GJ in the present study are comparable to those recently reported for *S. cerevisiae* grown on standard yeast media in the presence of the squalene epoxidase inhibitor, terbinafine [27]. However, in that study a terbinafine concentration of 300 μM was required to produce the optimum squalene titre (20.70 ± 1.00 mg L^−1^). In our work, a comparable squalene titre was achieved by repressing overall *ERG1* gene transcription with just 0.025 μg mL^−1^ (0.05 μM) doxycycline (Table 4). In addition to its sensitivity, use of the promoter system to attenuate squalene epoxidase synthesis at the *ERG1* gene level circumvents the potential to select for protein-level mutations that conserve squalene epoxidase function (that is, normal ergosterol biosynthesis) in yeast cultures treated with protein inhibitors (for example, terbinafine [27]). Terbinafine resistant *S. cerevisiae* harbouring single amino acid substitutions in the Erg1 protein (either L251F, F402L, F420L or P430S) have already been reported [49]. The economic feasibility of using the *tet0*_*7*_*-CYC1* promoter system to harness squalene production on an industrial scale is currently being assessed. Owing to the demand and high commercial value of squalene, it is anticipated that the costs of doxycycline supplementation would likely be offset by those recovered from squalene production. The design and use of alternative yeast promoter systems (such as *GAL1, CUP1* and *MET3* [[50] for summary]) is possible. However, it is important that the promoter of choice does not have an effect on yeast physiology, and that is what is so attractive about the doxycycline system.

### Grass biomass and microbial bioprocess engineering

Grass biomass comprises several fractions (for example, water-soluble sugars, fructans, hemicellulose, cellulose [39]) that could be used as substrates for the production of biofuels and other value-added bio-based compounds. In the present study, 55% of the original fructan was still present in grass juice at the end of the fermentation experiments (Table 3). The enzymatic hydrolysis of fructans in grass juice prior to yeast fermentation experiments has already been reported [37] and recombinant yeast that can simultaneously saccharify and ferment grass fructans to ethanol has recently been achieved [38]. Because simultaneous saccharification and fermentation requires fewer steps than enzyme addition, we envisage further modification of YUG37-*ERG1* to enable utilisation of fructan; this could enhance the efficiency and yields of ethanol and squalene produced from grass juice. Use of alternative yeast species (for example, *S. kudriavzevii* [51]) and existing recombinant industrial strains (for example, see [52]) that can utilise alternative substrates and agro-industrial feedstocks for the production of squalene and ethanol is also of interest.

## Conclusion

Results from this study clearly demonstrate proof of principle that squalene production can be harnessed in *S. cerevisiae* by repressing *ERG1* gene transcription. The potential to co-produce ethanol and squalene (and/or additional bio-based products) from a single feedstock using yeast is realistic and warrants further investigation.

## Abbreviations

ATP: adenosine triphosphate
ERG1: squalene epoxidase
GJ: grass juice
GRAS: generally regarded as safe
MgSO_4_: magnesium sulphate
OD: optical density
YPD: yeast-peptone-dextrose
